# Predictive Clinical Factors in the Diagnosis of Gastrointestinal Kaposi's Sarcoma and Its Endoscopic Severity

**DOI:** 10.1371/journal.pone.0046967

**Published:** 2012-11-30

**Authors:** Naoyoshi Nagata, Takuro Shimbo, Hirohisa Yazaki, Naoki Asayama, Junichi Akiyama, Katsuji Teruya, Toru Igari, Norio Ohmagari, Shinichi Oka, Naomi Uemura

**Affiliations:** 1 Department of Gastroenterology and Hepatology, National Center for Global Health and Medicine, Tokyo, Japan; 2 Clinical Research and Informatics, International Clinical Research Center Research Institute, National Center for Global Health and Medicine, Tokyo, Japan; 3 Division of AIDS Clinical Center, National Center for Global Health and Medicine, Tokyo, Japan; 4 Department of Clinical Pathology, National Center for Global Health and Medicine, Tokyo, Japan; 5 Department of Infectious Disease, National Center for Global Health and Medicine, Tokyo, Japan; 6 Department of Gastroenterology and Hepatology, National Center for Global Health and Medicine, Kohnodai Hospital, Chiba, Japan; University of Alabama at Birmingham, United States of America

## Abstract

**Background:**

The diagnosis of gastrointestinal (GI) involvement in Kaposi's sarcoma (KS) is important to make because the need for treatment depends on the extent of the disease. Moreover, severe GI lesions can cause serious complications. Endoscopy with biopsy is an extremely useful method to diagnose GI-KS. However, determining the indications for endoscopy is difficult because KS can occur without GI symptoms or cutaneous KS. This study sought to clarify predictive clinical factors for GI-KS and its severity on endoscopy.

**Methodology/Principal Findings:**

A total of 1,027 HIV-infected patients who underwent endoscopy were analyzed. Sexual behavior, CD4 count, HIV RNA, history of highly active antiretroviral therapy (HAART), GI symptoms, and cutaneous KS were assessed. Endoscopic severity including bulky tumor, ulceration, and number of lesions were evaluated. Thirty-three patients had GI-KS and 46 patients cutaneous KS. Among the GI-KS patients, 78.8% (26/33) had no GI symptoms and 24.2% (8/33) had no cutaneous KS. Univariate analysis identified men who have sex with men (MSM), CD4 <100 cells/µL, HIV RNA ≥10,000 copies/mL, no history of HAART, and cutaneous KS were significantly associated with GI-KS. Among these factors, cutaneous KS was closely related to GI-KS on multivariable analysis. Among patients without cutaneous KS, MSM and CD4 count <100 cells/µL were the only independent clinical factors related to GI-KS. Bulky tumor was significantly associated with CD4 <100 cells/µL and large number of lesions was significantly associated with HIV-RNA ≥10,000 copies/mL.

**Conclusions:**

To diagnose GI-KS, clinical factors need to be considered before endoscopy. The presence of GI symptoms is not useful in predicting GI-KS. MSM and CD4 count <100 cells/µL are predictive factors among patients without cutaneous KS. Caution should be exercised especially in patients with low CD4 counts or high HIV viral loads as they are more likely to develop severe GI-KS lesions.

## Introduction

Kaposi's sarcoma (KS) is a rare type of cancer of the lymphatic and blood vessels that most commonly involves the skin [1]–[3]. KS is more prevalent in HIV-infected patients, especially among men who have sex with men (MSM) [2], [3]. Although the rate of AIDS-related KS has decreased dramatically since the introduction of highly active antiretroviral therapy (HAART) [4]–[6], KS remains the most common malignancy among patients with AIDS [7].

The diagnosis of visceral involvement of KS is important to make because the need for treatment and choice of treatment depend on the extent of the disease [4]–[11]. The gastrointestinal (GI) tract is a common site of visceral involvement [12]–[16]. Endoscopy with biopsy is extremely useful for diagnosing GI-KS and is usually indicated for patients with GI symptoms and the presence of cutaneous KS [17], [18]. However, GI-KS can occur without GI symptoms [19], [20] and in the absence of cutaneous disease [20], [21]. Moreover, few studies have investigated the clinical factors of GI-KS [19]–[21] and most of those have been case series or case reports without control subjects. Therefore, the indications for endoscopy to detect GI-KS in patients with HIV/AIDS, especially those without GI symptoms or cutaneous disease, have been difficult to determine.

Endoscopically, GI-KS can vary from flat maculopapular or polypoid masses to severe lesions. The latter can cause serious complications such as hemorrhage, perforation, and obstruction and may require emergent treatment [14], [22]–[26]. However, there are no reports to date on the predictive clinical factors for finding severe GI-KS lesions on endoscopy.

In Japan, screening endoscopy is frequently performed for the early detection of malignant or premalignant lesions, even as part of the examination for patients who are asymptomatic. In this study, we performed endoscopy in a large number of HIV-infected patients with or without GI symptoms and cutaneous involvement.

## Methods

### Objectives

We conducted a case-control study to identify predictive clinical factors for diagnosing GI-KS, especially among patients without GI symptoms and cutaneous disease. We also assessed macroscopic appearance in detail looking for predictors of severe GI-KS lesions on endoscopy.

### Participants

We recruited 1,064 HIV-infected patients who had undergone endoscopy between 2003 and 2009 at the National Center for Global Health and Medicine (NCGM), a 900-bed hospital located in the Tokyo metropolitan area and the largest referral center for HIV/AIDS in Japan. We excluded patients who had received endoscopy for follow-up evaluation shortly after treatment for GI disease.

### Ethics statement

The institutional review board at NCGM approved this study. All patients from whom clinical samples were obtained during endoscopy or biopsy had provided written informed consent prior to endoscopy. No ethical problems exist with regard to the publication of this manuscript. We used anonymized data from patient medical records.

### Clinical factors

Before endoscopy, we routinely enter “purposes of the inspection” into the electronic endoscopic database. Purposes include examination for symptoms, screening for malignant or premalignant lesions, and follow up for endoscopic procedure or surgery. GI symptoms were assessed by the physician who interviewed each patient in detail. Those without GI symptoms and negative screening endoscopy were considered to be symptom-free.

CD4 cell counts were checked within 1 week of endoscopy. We categorized CD4 cell counts into four groups: ≥300 cells/µL; 200–299 cells/µL; 100–199 cells/µL; and <100 cells/µL. HIV-RNA viral load (VL) as determined by real-time quantitative polymerase chain reaction (PCR) was reviewed within 1 month of endoscopy. The minimum detection level was 40 copies/mL of plasma. A positive result for real-time HIV RNA was defined as ≥40 copies/mL. HIV-RNA VL was categorized into four groups: VL ≤40 copies/mL (normal range); 40<VL≤10,000 copies/ml; 10,000<VL≤100,000 copies/mL; and VL>100,000 copies/mL.

History of HAART was collected from medical records prior to endoscopy and was categorized into five groups according to duration of administration: without history of HAART; duration ≤6 months; 6 months<duration ≤1 year; 1 year<duration ≤5 years; duration >5 years.

HIV infection route was determined by the medical staff who interviewed each patient on the first visit to our hospital and classified into five categories: MSM, heterosexual, hemophiliac, injection drug user, and unknown. We defined sexual behavior as MSM or heterosexual. Patients who were not homosexual or bisexual were regarded as heterosexual.

### Diagnosis of GI-KS

**Figure 1:**
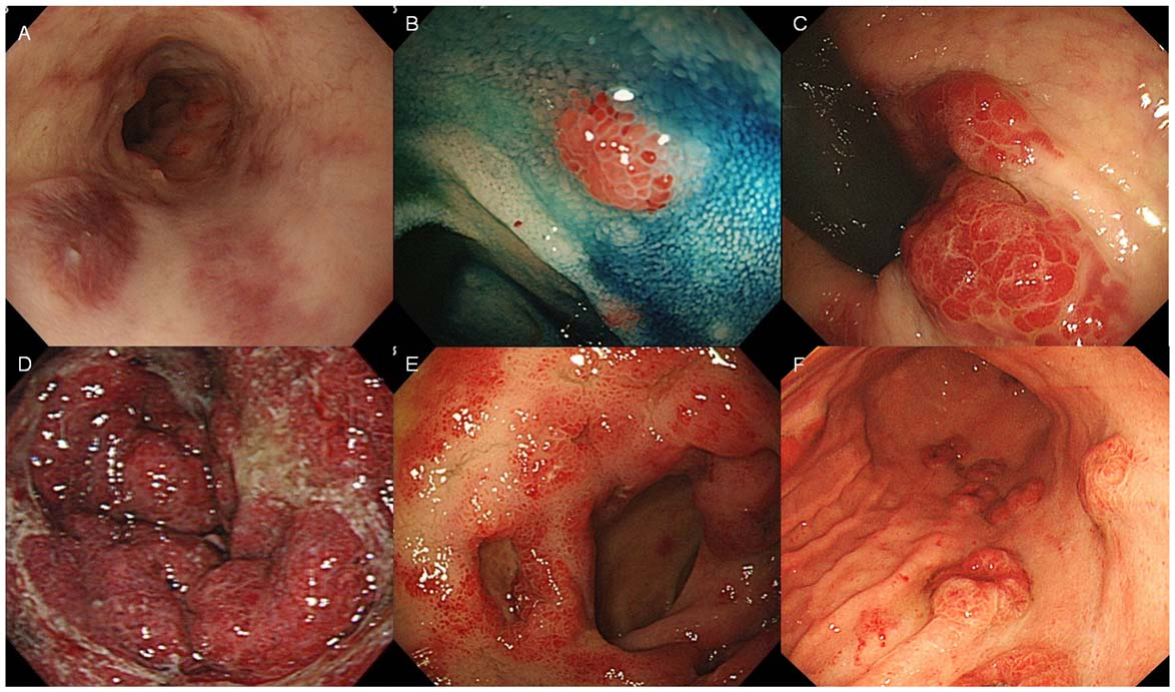
Gastrointestinal Kaposi's sarcoma on endoscopy. **A**) Dark-reddish flat lesions in the esophagus. **B**) Chromoendoscopy with indigo carmine dye showed a polypoid nodule in the terminal ileum. **C**). Submucosal lesions in the rectum. **D**) Bulky tumor surrounding the antrum of the stomach and causing pyloric stenosis. **E**) Circumferential flat lesions with ulceration in the duodenum. **F**) Multiple nodules in the stomach.

We performed biopsy when abnormal findings were encountered on upper or lower endoscopy. If we performed both upper and lower endoscopy in the same individual, this was defined as one case. GI-KS was suspected based on endoscopic appearance, such as the presence of submucosal nodules, polypoid nodules, or deep-red mucosa (Fig. 1A–C), as previously reported [16], [19], [27]. Endoscopic severity was evaluated in terms of appearance, including size, ulceration, and number of lesions. Regarding size, we defined bulky tumors (Fig. 1D) as circumferential or obstructive lesions and small tumors as all other cases [23]. Ulceration was defined endoscopically as a distinct, visible crater >5 mm in diameter with a slough base (Fig. 1E). The number of lesions was classified into two groups: large number (≥10) (Fig. 1F) or small number (<10).

**Figure 2:**
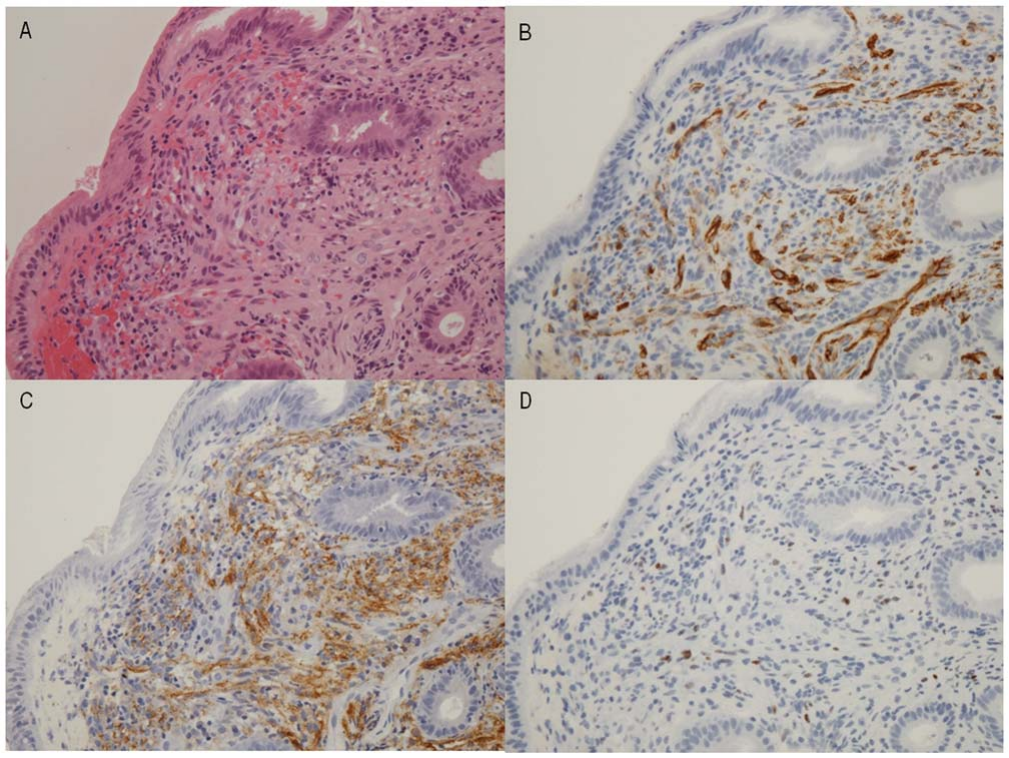
Pathological features of GI-KS. **A**) Spindle cell proliferation found in the submucosa on hematoxylin and eosin (HE) staining. **B**) Immunohistochemical staining revealing strong expression of CD34. **C**) Immunohistochemical staining revealing expression of D2–40. Vascular gaps are lined with endothelial cells on staining for CD34 and D2–40. **D**) Some endothelial cells are positive for human herpes virus 8 (HHV-8).

GI-KS was defined as the presence of proliferating spindle cells in biopsy specimens as seen on hematoxylin and eosin (HE) staining (Fig. 2A). Spindle cells were consistently positive on immunohistochemical staining for D2–40 (Fig. 2B), CD34 (Fig. 2C), and HHV-8 (Fig. 2D), as previously reported [28], [29]. We assessed the presence of lesions in the esophagus, stomach, duodenum, terminal ileum, colon, and rectum. Before endoscopy, we examined all patients for cutaneous lesions of KS.

### Statistical analysis

After summarizing the descriptive patient characteristics, to identify predictive clinical factors for diagnosing GI-KS, we calculated odds ratios (ORs) and 95% confidence intervals (CIs). The relationship between GI-KS and categories of CD4 cell count, HIV-RNA VL, and HAART duration were evaluated using the chi-square test for linear trends. For multivariable analysis, we used a multiple logistic regression model that included all factors showing values of p<0.1 on univariate analysis. Exact logistic regression was also used if the number in a cell was 0. A final model was then developed by backward selection of factors showing values of p<0.10.

The relationships between endoscopic severity of GI-KS and clinical factors were also evaluated using the chi-square test. Values of p<0.10 were considered significant. All statistical analysis was performed using Stata version 10 software (StataCorp LP, College Station, TX).

## Results

### Participants

Of the 1,064 potential study subjects recruited who underwent endoscopy, we excluded 37 who underwent endoscopy for follow-up evaluation shortly after treatment for GI diseases. Ultimately, the remaining 1,027 patients were selected for data analysis.

### Baseline characteristics

Characteristics of the 1,027 patients with HIV are shown in Table 1. Median age was 44 years (interquartile range [IQR], 36–56 years). Patients were predominantly male (91.8%).

**Table 1:** Patient characteristics.

	All (n = 1,027)	With GI-KS (n = 33)	Without GI-KS (n = 994)
Age, median (IQR)*	44 (36, 56)	44 (36, 56)	45 (35, 55)
Sex (male), n (%)	943 (91.8%)	33 (100%)	910 (91.6%)
HIV infection route, n (%)			
MSM	688 (67.0%)	31 (93.9%)	657 (66.1%)
Heterosexual	175 (17.0%)	2 (6.1%)	173 (17.4%)
Hemophiliac	140 (13.6%)	0	140 (14.1%)
Drug-user	2 (0.2%)	0	2 (0.2%)
Unknown	22 (2.2%)	0	22 (2.2%)
CD4 cell count (cells/µL)			
≥300	424 (41.3%)	1 (3.0%)	423 (42.6%)
200–299	155 (15.1%)	3 (9.1%)	152 (15.3%)
100–199	193 (18.8%)	8 (24.2%)	185 (18.6%)
<100	255 (24.8%)	21 (63.7%)	234 (23.5%)
HIV RNA (copies/mL)			
VL≤40 (normal range)	533 (51.9%)	4 (12.1%)	529 (53.2%)
40<VL≤10,000	176 (17.1%)	4 (12.1%)	172 (17.3%)
10,000<VL≤100,000	151 (14.7%)	7 (21.1%)	144 (14.5%)
VL>100,000	167 (16.3%)	18 (54.6%)	149 (15.0%)
History of HAART, n (%)			
Without history of HAART	288 (28.0%)	18 (54.6%)	270 (27.2%)
Duration ≤6 months	113 (11.0%)	8 (24.2%)	105 (10.6%)
6 months<duration≤1 yr	75 (7.3%)	7 (21.2%)	68 (6.8%)
1 yr<duration≤5 yrs	67 (6.5%)	0	67 (6.7%)
Duration >5 yrs	484 (47.1%)	0	484 (48.7%)
GI symptoms, n (%)			
Without	659 (64.2%)	26 (78.8%)	633 (63.7%)
With	368 (35.8%)	7 (21.2%)	361 (36.3%)
Cutaneous KS			
Without	981 (95.5%)	8 (24.2%)	973 (97.9%)
With	46 (4.5%)	25 (75.8%)	21 (2.1%)

Abbreviations: IQR, interquartile range; MSM, men who have sex with men; VL, viral load; yrs, years; GI, gastrointestinal.

Routes of HIV infection included MSM (67.0%), heterosexual (17.0%), hemophilia (13.6%), drug usage (0.2%), and unknown (2.1%). Median CD4 count was 239 cells/µL (IQR, 100–406 cells/µL). Median HIV-RNA VL was <40 copies/mL (IQR, <40–33,000 copies/mL). A total of 739 patients (72.0%) had received HAART.

GI symptoms were noted in 368 patients (35.8%) as follows: appetite loss (n = 6); throat pain (n = 8); dysphagia (n = 11); reflux or heartburn (n = 22); epigastric pain (n = 87); nausea or vomiting (n = 36); hematemesis (n = 25); tarry stool (n = 43); hematochezia (n = 54); diarrhea (n = 87); distended abdomen (n = 2); and lower abdominal pain (n = 17).

### Characteristics of GI-KS

Of the 1,027 patients, 33 (3.2%) were diagnosed with GI-KS (Table 1). GI lesions were found in the esophagus (n = 10), stomach (n = 20), duodenum (n = 17), terminal ileum (n = 7), colon (n = 13), and rectum (n = 9). Of the patients with GI-KS, 78.8% (26/33) had no GI symptoms and 24.2% (8/33) had no cutaneous KS (Table 1).

Five of eight GI-KS patients without cutaneous involvement underwent HAART for a mean duration of 1.84 months.

### Predictive clinical factors of GI-KS

Univariate analysis identified MSM, CD4 count <100 cells/µL, HIV RNA VL ≥10,000 copies/mL, no history of HAART, and the presence of cutaneous KS as significant clinical factors for the development of GI-KS (Table 2).

**Table 2:** Predictive clinical factors for GI-KS on uni- and multivariable analysis.

	All (n = 1.027)	Without GI symptoms (n = 659)	Without cutaneous KS (n = 981)
Univariate analysis	Odds ratio (95%CI)	Odds ratio (95%CI)	Odds ratio (95%CI)
Age (years)			
<40	1 (referent)	1 (referent)	1 (referent)
≥40	0.84 (0.41–1.72)	1.00 (0.43–2.34)	0.54 (0.13–21.8)
Sex			
Female	1 (referent)	1 (referent)	1 (referent)
Male	4.33 (0.76–∞)†	3.70 (0.64–∞)†	1.04 (0.16–∞)†
Sexual behavior			
Heterosexual	1 (referent)	1 (referent)	1 (referent)
MSM	7.95 (1.89–33.4)*	5.67 (1.33–24.2)**	5.78 (0.89–∞)† ***
CD4 cell count (cells/µL)			
≥100	1 (referent)	1 (referent)	1 (referent)
<100	5.68 (2.75–11.7)*	4.43 (1.99–9.85)*	10.3 (2.06–51.2)*
HIV RNA (copies/mL)			
<10,000	1 (referent)	1 (referent)	1 (referent)
≥10,000	7.40 (3.30–16.6)*	8.52 (3.37–21.6)*	1.49 (0.35–6.29)
History of HAART			
Without	1 (referent)	1 (referent)	1 (referent)
With	0.31 (0.15–0.63)*	0.26 (0.12–0.58)*	0.59 (0.14–2.49)
GI symptoms			
Without	1 (referent)	NA	1 (referent)
With	0.47 (0.20–1.10)***	NA	1.02 (0.24–4.30)
Cutaneous KS			
Without	1 (referent)	1 (referent)	NA
With	144.8 (58.5–358.2)*	128.7 (44.1–376.1)*	NA
**Multivariable analysis**	Odds ratio (95%CI)	Odds ratio (95%CI)	Odds ratio (95%CI)
MSM			5.18 (0.79–∞)† ***
CD4 count <100 cells/µL			9.55 (1.69–97.7)† *
Cutaneous KS	144.8 (58.5–358.2)	128.7 (44.1–376.1)*	NA

† : Analysis by exact logistic regression.

* p<0.01.

** p<0.05.

*** p<0.1.

A final model of multivariable analysis was developed by backward selection of factors showing values of p<0.05.

Abbreviations: GI-KS, gastrointestinal Kaposi's sarcoma; MSM, men who have sex with men; HAART, highly active antiretroviral therapy; NA, not applicable.

As the CD4 count decreased (≥300; 200–299; 100–199; and <100 cells/µL), occurrence of GI-KS increased significantly (p<0.01 for trend in odds, Table 1). As HIV RNA viral load increased (VL≤40; 40<VL≤10,000; 10,000<VL≤100,000; and VL>100,000 copies/ml), the occurrence of GI-KS increased significantly (p<0.01 for trend in odds, Table 1). Multivariable analysis showed cutaneous KS (OR, 144.8, 95%CI, 58.5–358.2, p<0.01) was the only independent clinical factor related to GI-KS.

### Predictive clinical factors of GI-KS in patients without GI symptoms

Univariate analysis identified MSM, CD4 count <100 cells/µL, HIV RNA VL≥10,000 copies/mL, no history of HAART, and presence of cutaneous KS as significant clinical factors for the development of GI-KS (Table 2). Multivariable analysis showed cutaneous KS (OR, 128.7, 95%CI, 44.1–376.1, p<0.01) was the only independent clinical factor related to GI-KS.

### Predictive clinical factors of GI-KS in patients without cutaneous KS

Univariate analysis identified MSM and CD4 count <100 cells/µL as significant clinical factors for the development of GI-KS (Table 2). Multivariable analysis showed MSM (OR, 5.18, 95%CI, 0.79–∞, p = 0.09) and CD4 count <100 cells/µL (OR, 9.55, 95%CI, 1.69–97.7, p<0.01) were the only independent clinical factors related to GI-KS.

### Predictive clinical factors for endoscopic severity of GI-KS

Endoscopic severity was found in the form of bulky tumors (n = 10), ulcerous lesions (n = 11), and multiple lesions (n = 9). Relationships between endoscopic severity of GI-KS and clinical factors are shown in Table 3. In the analysis of GI-KS patients, endoscopic severity in the form of bulky tumors was found to be associated with CD4 cell count <100 cells/µL (p = 0.04). Endoscopic severity in the form of multiple lesions was found to be associated with HIV RNA VL≥10,000 copies/mL (p<0.05). No significant difference was noted in the presence of cutaneous KS between the mild groups and the severe group on endoscopy.

**Table 3:** Relationship between endoscopic severity of GI-KS and clinical factors (n = 33).

Factor	GI-KS with small tumor (n = 23)	GI-KS with bulky tumor (n = 10)	GI-KS without ulcer (n = 22)	GI-KS with ulcer (n = 11)	GI-KS small number (n = 24)	GI-KS large number (n = 9)
Age (yrs) ≥40	60.9%	60.0%	68.2%	45.5%	58.3%	66.7%
Sex (male)	100%	100%	100%	100%	100%	100%
Sexual behavior (MSM)	91.3%†	100%†	95.5%∫	90.9%∫	95.8%¶	88.9%¶
CD4 cell counts <100 cells/µL	52.2%†	90.0%†	59.1%∫	72.2%∫	62.5%¶	66.7%¶
HIV RNA ≥10,000 copies/mL	73.9%†	80.0%†	68.2%∫	90.9%∫	66.7%¶	100%¶
History of HAART	47.8%†	40.0%†	45.5%∫	45.5%∫	45.8%¶	44.4%¶
With GI symptoms	21.7%	20.0%	22.7%	18.2%	25.0%	11.1%
With cutaneous KS	21.7%†	30.0%†	77.3%∫	72.7%∫	79.2%¶	66.7%¶

† : Analysis by chi-square test between GI-KS with small tumor and bulky tumor, MSM (p = 0.34), CD4 (p<0.05), HIV-RNA (p = 0.71), history of HAART (p = 0.68), and presence of cutaneous KS (p = 0.61).

∫ : Analysis by chi-square test between GI-KS with small tumor and bulky tumor, MSM (p = 0.61), CD4 (p = 0.44), HIV-RNA (p = 0.15), history of HAART (p = 1.00), and the presence of cutaneous KS (p = 0.77).

¶ : Analysis by chi-square test between GI-KS with small tumor and bulky tumor, MSM (p = 0.61), CD4 (p = 0.83), HIV RNA (p<0.05), history of HAART (p = 0.94), and presence of cutaneous KS (p = 0.46).

Abbreviations: GI-KS, gastrointestinal Kaposi's sarcoma; MSM, men who have sex with men; HAART, highly active antiretroviral therapy.

## Discussion

Endoscopy is clearly a valuable diagnostic method for identifying GI-KS, but it is not recommended for all HIV-infected patients because of considerations of cost and invasiveness. The present study therefore sought to answer which HIV-infected patients need endoscopy to detect visceral KS.

We found that MSM, low CD4 (<100 cells/µL), high HIV RNA VL (>10,000 copies/mL), no history of HAART, and presence of cutaneous KS were predictive clinical factors for GI-KS on univariate analysis. Our findings are consistent with past studies on HIV-infected patients from Western countries that showed an association between clinical factors and cutaneous KS or visceral KS [2], [2], [4]–[7], [9]–[13].

Endoscopy is usually considered to be indicated for GI-KS diagnosis in patients who have GI symptoms [17], [18]. However, GI-KS can reportedly be detected even in patients without GI symptoms [19], [20]. Indeed, we found that 79% of the patients we identified with GI-KS were asymptomatic. Both uni- and multivariable analysis showed that the presence of GI symptoms is not useful in predicting GI-KS. Furthermore, predictive factors for GI-KS were unchanged among patients without GI symptoms.

Previous studies have shown that KS occurs more commonly among MSM with AIDS than among heterosexual individuals with AIDS [2], [7], [8]. However, the true sexual behavior of a patient might not be able to be ascertainable in interviews, as was attempted in this study. Some MSM patients may well have been included among the unknown cases of non-GI-KS patients, in whom the OR of sexual behavior would be difficult to evaluate.

HAART is known to represent a highly effective treatment for GI-KS, and can improve the immune status of patients [4]–[6], [9]–[11]. The present study found that a history of long-term administration of HAART reduced the occurrence of GI-KS.

It has previously been shown that patients with KS typically have a low CD4 cell count (<150 cells/µL) and a high HIV RNA VL (>10,000 copies/mL) [9]–[11]. However, CD4 levels and HIV-RNA VLs have yet to be fully investigated in GI-KS patients. The present study demonstrated that the prevalence of GI-KS tended to increase significantly with low CD4 cell count and with high HIV RNA VL. The presence of GI involvement with KS may vary according to immune status.

KS manifests primarily as a cutaneous disorder, with visceral involvement considered to occur subsequently [2], [12]. In this study, the presence of cutaneous KS was found to be closely related to GI-KS on uni- and multivariable analysis. These results suggest that endoscopy may be indicated for patients with cutaneous KS.

It has been reported that GI-KS can occur in the absence of cutaneous disease [20], [21]. Thus, we assessed the clinical factors among patients without cutaneous disease. We found that MSM and CD4 count <100 cells/µL were the only independent clinical factors related to GI-KS. Our results represent the first confirmation of this finding evaluated in a case-control study.

We investigated the possibility that HAART administration led to the disappearance of cutaneous KS prior to the diagnosis of GI-KS in patients without cutaneous involvement. In fact, five of eight GI-KS patients without cutaneous KS had a history of HAART. However, the mean duration of the administration was less than 2 months, and it is difficult to imagine that cutaneous KS disappeared from the whole body in such a short period of time. Therefore, it is unlikely that HAART administration had any involvement in the absence of cutaneous KS in these GI-KS patients.

In this study, we assessed endoscopic severity such as tumor bulk, ulceration, and multiple lesions as these may cause obstruction, hemorrhage, and perforation [14], [22]–[26]. We found, for the first time, that CD4 count (<100 cells/µL) or high HIV RNA VL (≥10,000 copies/mL) were key clinical factors to predict severe GI lesions on endoscopy.

A key limitation of this study was the single-center, retrospective nature of the investigation. When considering indications for endoscopy, a randomized controlled study is required to identify whether endoscopy can prevent the development of severe GI complications in HIV-infected patients, particularly among those with a low CD4 count. Second, the number of GI-KS patients was relatively small, especially those without cutaneous KS. The statistical power of the study might thus have been low due to the small number of cases.

### Conclusions

To diagnose GI-KS, clinical factors need to be considered before endoscopy is undertaken. Presence of GI symptoms is not useful in predicting GI-KS. The presence of cutaneous KS, MSM sexual behavior, low CD4 count (<100 cells/µL), high HIV RNA VL, and no history of HAART are predictive factors for GI-KS. Even if patients have no cutaneous KS, endoscopy may be suitable for patients with MSM and low CD4 count (<100 cells/µL). Caution should be exercised especially in patients with a low CD4 count (<100 cells/µL) or high HIV RNA VL (≥10,000 copies/mL) as they are more likely to develop severe GI-KS lesions. This diagnostic strategy could facilitate early diagnosis of GI-KS.
